# Prevalence and factors associated with multimorbidity in adults in Brazil, according to sex: a population-based cross-sectional survey

**DOI:** 10.3389/fpubh.2023.1193428

**Published:** 2023-06-02

**Authors:** Cristina Camargo Pereira, Charlise Fortunato Pedroso, Sandro Rogério Rodrigues Batista, Rafael Alves Guimarães

**Affiliations:** ^1^Institute of Tropical Pathology and Public Health, Federal University of Goiás, Goiânia, Brazil; ^2^Department of Internal Medicine, School of Medicine, Federal University of Goiás, Goiânia, Brazil; ^3^Federal District Health Department, Brasília, Brazil; ^4^Faculty of Nursing, Federal University of Goiás, Goiânia, Brazil

**Keywords:** multimorbidity, multiple chronic conditions, chronic disease, risk factors, public health

## Abstract

**Introduction:**

Multimorbidity, defined as the coexistence of two or more chronic diseases in the same individual, represents a significant health challenge. However, there is limited evidence on its prevalence and associated factors in developing countries, such as Brazil, especially stratified by sex. Thus, this study aims to estimate the prevalence and analyze the factors associated with multimorbidity in Brazilian adults according to sex.

**Methods:**

Cross-sectional population-based household survey carried out with Brazilian adults aged 18 years or older. The sampling strategy consisted of a three-stage conglomerate plan. The three stages were performed through simple random sampling. Data were collected through individual interviews. Multimorbidity was classified based on a list of 14 self-reported chronic diseases/conditions. Poisson regression analysis was performed to estimate the magnitude of the association between sociodemographic and lifestyle factors with the prevalence of multimorbidity stratified by sex.

**Results:**

A total of 88,531 individuals were included. In absolute terms, the prevalence of multimorbidity was 29.4%. The frequency in men and women was 22.7 and 35.4%, respectively. Overall, multimorbidity was more prevalent among women, the older people, residents of the South and Southeast regions, urban area residents, former smokers, current smokers, physically inactive, overweight, and obese adults. Individuals with complete high school/incomplete higher education had a lower prevalence of multimorbidity than those with higher educational level. The associations between education and multimorbidity differed between sexes. In men, multimorbidity was inversely associated with the strata of complete middle school/incomplete high school and complete high school/incomplete higher education, while in women, the association between these variables was not observed. Physical inactivity was positively associated with a higher prevalence of multimorbidity only in men. An inverse association was verified between the recommended fruit and vegetable consumption and multimorbidity for the total sample and both sexes.

**Conclusion:**

One in four adults had multimorbidity. Prevalence increased with increasing age, among women, and was associated with some lifestyles. Multimorbidity was significantly associated with educational level and physical inactivity only in men. The results suggest the need to adopt integrated strategies to reduce the magnitude of multimorbidity, specific by gender, including actions for health promotion, disease prevention, health surveillance and comprehensive health care in Brazil.

## 1. Introduction

Chronic noncommunicable diseases (NCDs) are one of the major challenges faced by governments and global health systems. These diseases account for approximately 71% of global mortality, especially cardiovascular diseases, cancers, chronic respiratory diseases, and diabetes (1). NCDs are responsible for 41 million deaths annually, most in low- and middle-income countries (1).

Multimorbidity is the coexistence of two or more chronic diseases/conditions in an individual (2); however, some studies have used other definitions, such as ≥3, ≥4, and ≥ 5 (3). The most common chronic diseases used to define multimorbidity are cardiovascular diseases, hypertension, diabetes, and dyslipidemias (3). Previous systematic reviews identified the prevalence of multimorbidity from 33.1 to 43.0% (3–5). A meta-analysis found a pooled multimorbidity prevalence of 42.4% in adults (3). Multimorbid individuals have a higher probability of hospitalization, disability, and death than those with a single chronic disease/condition (6, 7). Multimorbidity is also related to declining physical function (8), worse quality of life (9), and higher socioeconomic costs for countries, individuals, and families (10).

In Brazil, multimorbidity is a serious challenge for public health systems. A recent systematic review reported a substantial variation in the estimated prevalence of multimorbidity from 16.8 to 93.4% (5). National-level assessment using data from the Brazil National Household Sample Survey and the National Health Survey (NHS) showed an increase in multimorbidity prevalence from 17.1 to 21.0% between 1998 and 2013 (8).

Multiple factors are associated with multimorbidity. Systematic reviews have shown a higher prevalence in older populations, women (3), and people with lower socioeconomic status and education (11). Several NCD risk factors, including cigarette smoking, alcohol use, decreased physical activity, and unhealthy diet, are associated with various diseases used to calculate multimorbidity (12). Prior studies have reported a positive association between multimorbidity and smoking (current or former smoker) and physical inactivity (13–15). However, healthy eating indicators are controversial in the literature. Data show the association of protection (16), risk (17, 18), and no association (19, 20). Thus, it remains unclear which risk factors most influence multimorbidity.

Despite the existing literature, the epidemiology and factors associated with multimorbidity are still poorly understood, especially in developing countries like Brazil (3). Furthermore, few studies have explored the prevalence according to sociodemographic and lifestyle variables. Many also do not include potential interactions in regression models, such as between race/skin color, education, and geographic region. Investigations of associated factors stratified by sex are also limited. Studies indicate that factors related to multimorbidity may differ between sexes (11, 21).

Most previous studies have found a higher prevalence of multimorbidity among women (4, 19, 21, 22), but not all studies have reported this (23–25). In almost all countries of the world, women have a higher life expectancy than men (26), they are more often affected by a series of non-fatal chronic diseases that negatively affect their quality of life and daily physical capacity (27). Thus, studies on multimorbidity require greater knowledge about its prevalence, the diseases involved and their relationship with possible gender differences. In Brazil, the prevalence of multimorbidity by gender has not been well documented in large populations.

Identifying multimorbidity risk factors stratified by sex is essential to effectively establish specific strategies for disease prevention, health promotion, and integrated care, particularly in a scenario of increased demand for health services due to population aging (28). The analysis of multimorbidity patterns can contribute to the planning of interventions to achieve Sustainable Development Goal 3 (Health and Wellbeing) and monitoring the Global Action Plan for the Prevention and Control of NCDs (29) and Strategic Plan for Tackle Chronic Diseases and Noncommunicable Diseases of the Brazilian Ministry of Health (30). Thus, this study aims to estimate the prevalence and analyze the factors associated with multimorbidity in Brazilian adults according to sex.

## 2. Materials and methods

### 2.1. Study design

Cross-sectional population-based study using data from the 2019 National Health Survey (NHS). The NHS is a household-based nationwide survey conducted by the Ministry of Health in partnership with the Oswaldo Cruz Foundation and the Brazilian Institute of Geography and Statistics (IBGE). One of the core objectives of the NHS is to outline the health situation and lifestyle behaviors of the Brazilian population, including the assessment of NCD patterns. The NHS is representative of urban and rural areas, large regions of Brazil, federative units, state capitals, and metropolitan regions (31).

### 2.2. Context

The 2019 NHS was carried out in the 27 federative units of Brazil distributed in five macro-regions (32). In 2019, the country had 5,570 municipalities and a total population of 210,147,125 inhabitants with a Human Development Index of 0.765, Gross Domestic Product (GDP) per capita of BRL 35,161.70, and household income per capita of BRL 1,439 (33).

### 2.3. Participants

The target population of the NHS consisted of individuals aged 15 years and older residing in permanent private households, which are built for the sole purpose of housing. Households located in special census tracts or sparsely populated areas were excluded from the sample such as indigenous villages, barracks, military bases, lodgings, campsites, prison units, long-stay institutions for the older people, comprehensive care facilities for children and adolescents, convents, hospitals, settlement project farming and quilombola communities (31). In the present study, participants aged 18 years and older were included.

The sampling strategy consisted of a three-stage conglomerate plan: (i) selection of Primary Sampling Units (PSU) constituted by census tracts, defined as the territorial unit formed by a continuous area located in a single urban or rural setting, with a defined dimension and number of households from the Geographic Operational Base of the 2010 Demographic Census of Brazil; (ii) selection of Secondary Sampling Units including households in each PSU, and; (iii) selection of Tertiary Sampling Units including individuals aged 15 or over who answered the research questionnaire. All individuals drawn from permanent private households were included. The three stages were performed through simple random sampling (31).

### 2.4. Sample size

The sample size defined for the 2019 NHS considered the desired accuracy for estimating the main risk and protective factors for NCDs, disease prevalence, and access to health services, among other variables. The average values and variances of the indicators were included, in addition to a 20% non-response rate. The sample was estimated at 108,525 households, and data were collected from 94,114 households, with a 6.4% non-response rate (31). In this study, the sample consisted of 88,531 adults.

### 2.5. Data collection

Data collection occurred during household visits from August 2019 to March 2020. The questionnaire used was validated by specialists and applied by trained professionals using mobile data collection devices for the interviews (31).

### 2.6. Variables

#### 2.6.1. Dependent variable

The dependent variable was multimorbidity, defined as the coexistence of ≥2 chronic diseases/conditions in an individual (2) using a list that includes one chronic condition and 13 self-reported chronic diseases: chronic back problem, hypertension, hypercholesterolemia, diabetes, arthritis or rheumatism, asthma or asthmatic bronchitis, work-related musculoskeletal disorders (WMSDs), cancer, cardiovascular diseases, stroke, chronic kidney failure, and chronic lung disease (such as pulmonary emphysema, chronic bronchitis or Chronic Obstructive Pulmonary Disease—COPD), depression and mental disorder. In order to assess people’s self-reports of chronic illnesses and conditions, participants answered a series of questions (Supplementary Table S1).

#### 2.6.2. Independent variables

The independent variables included were: sociodemographics [sex, age group (34), self-declared race/skin color (35), education (36), living with a spouse/partner, geographic region (37), and area of residence (37)]; lifestyle [tobacco use (38), alcohol abuse (39), leisure-time physical activity (40), recommended consumption of fruits and vegetables (41), ultra-processed food consumption (41), regular consumption of soft drinks or artificial juices (41), and nutritional status (42)]. Questions, answer options, operational definitions and, analysis categories are in Supplementary Table S2.

### 2.7. Statistical analysis

The data collected using mobile was stored in a database. The participant’s identification data has been deleted and replaced by a unique code. Data quality, such as the number of missing data, was checked. All variables were treated and categorized if necessary. In addition, sample weights were calculated.

Statistical analysis was conducted using the STATA software (StataCorp LLC, version 15.0, College Station, TX, EUA). First, a descriptive analysis of the variables stratified by sex was performed, using relative frequencies (%) and their respective 95% confidence intervals (95% CI). Then, the prevalence of each chronic disease/condition by sex and age group was determined. Statistical differences between groups were verified using the chi-square test adjusted by the study design.

Subsequently, bivariate Poisson regression was used to predict the association between each independent variable and multimorbidity. The final models were reported as unadjusted Prevalence Ratios (PR) and respective 95% CI. Sociodemographic and lifestyle variables with value of *p* < 0.20 in the bivariate analysis were included in the multiple regression model. We also incorporated interaction terms between the following variables: age group versus geographic region; education versus self-reported race/skin color; geographic region versus self-reported race/skin color; education versus geographic region; and ultra-processed food consumption versus recommended fruit and vegetable consumption. These factors were included based on statistical significance among all interactions and/or the theory of potential interactions described in the literature. The results of this analysis were reported as adjusted PR (APR) and respective 95% CI. Analyzes were performed for the total sample and stratified by sex. *p*-values < 0.05 were considered statistically significant.

### 2.8. Ethical aspects

The NHS was approved by the Research Ethics Committee of the National Health Council under protocol number 3,529,376/2019. Written consent was obtained from all participants.

## 3. Results

### 3.1. Sample characteristics

A total of 88,531 participants were included. The mean age was 44.9 years (95% CI: 44.7–45.2, minimum 18 years and maximum 102 years), and most were women (53.2, 95% CI: 52.6–53.8).

According to Table 1, the majority were aged between 40 and 59 years old (35.3%), brown (43.8%), had completed high school or incomplete higher education (34.9%), reported living with a spouse (61.4%), in the Southeast region (43.4%), urban area (86.2%), and did not live in capitals or metropolitan regions (58.4%). Regarding lifestyle-related risk factors, 26.6% were former smokers, 17.1% reported binge drinking, most were physically inactive (69.9%), 13.0% regularly consumed fruits and vegetables, 14.3% consumed five or more ultra-processed food groups, and 32.9% regularly drink soda or artificial juice. Significant differences between men and women were observed for most variables, including those related to lifestyle.

**Table 1:** Sample description according to sex.

Variables	Total (*n* = 88,531)	Men (*n* = 41,662)	Women (*n* = 46,869)	*p*-value^§^
	%^†^ (95% CI)	%^†^ (95% CI)	%^†^ (95% CI)	
Age group (years)				
18–24	13.9 (13.4–14.4)	14.9 (14.1–15.6)	13.0 (12.3–13.7)	<0.001
25–39	29.2 (28.6–29.8)	30.1 (29.3–30.9)	28.4 (27.7–29.2)	
40–59	35.3 (34.7–35.9)	35.0 (34.2–35.9)	35.5 (34.7–36.3)	
≥60	21.6 (21.1–22.2)	20.0 (19.3–20.7)	23.0 (22.4–23.7)	
Self-reported race/skin color^*^				
White	43.3 (42.5–44.0)	42.5 (41.6–43.5)	43.9 (43.0–44.8)	0.085
Brown	43.8 (43.1–44.5)	44.3 (43.4–45.3)	43.3 (42.5–44.2)	
Black	11.5 (11.1–11.9)	11.6 (11.0–12.1)	11.4 (10.9–11.9)	
Others (yellow or indigenous)	1.5 (1.3–1.6)	1.6 (1.3–1.9)	1.4 (1.2–1.6)	
Education				
No education or incomplete middle school	34.8 (34.1–35.4)	35.5 (34.6–36.4)	34.1 (33.3–34.9)	<0.001
Complete middle school or incomplete high school	14.5 (14.1–14.9)	15.7 (15.1–16.4)	13.4 (12.8–14.0)	
Complete high school or incomplete higher education	34.9 (34.3–35.6)	34.6 (33.7–35.5)	35.2 (34.4–36.1)	
Complete higher education or more	15.8 (15.2–16.5)	14.2 (13.4–15.0)	17.3 (16.5–18.0)	
Living with a spouse/partner				
No	38.6 (38.0–39.3)	31.8 (30.8–32.7)	44.7 (43.8–45.5)	<0.001
Yes	61.4 (60.7–62.0)	68.2 (67.3–69.2)	55.3 (54.5–56.2)	
Geographic region				
North	7.8 (7.6–8.1)	8.1 (7.7–8.5)	7.6 (7.3–7.9)	0.312
Northeast	26.5 (25.9–27.0)	26.2 (25.5–27.0)	26.6 (26.0–27.3)	
Southeast	43.4 (42.6–44.2)	43.1 (42.0–44.3)	43.7 (42.8–44.6)	
South	14.7 (14.3–15.1)	14.9 (14.3–15.5)	14.5 (14.0–15.1)	
Midwest	7.6 (7.3–7.9)	7.7 (7.3–8.1)	7.5 (7.2–7.8)	
Area of residence				
Urban	86.2 (85.8–86.6)	84.3 (83.7–84.8)	87.9 (87.4–88.3)	<0.001
Rural	13.8 (13.4–14.2)	15.7 (15.2–16.3)	12.1 (11.7–12.6)	
Tobacco use				
Non-smoker	60.8 (60.2–61.4)	57.3 (56.4–58.2)	63.9 (63.1–64.7)	<0.001
Former smoker	26.6 (26.1–27.2)	26.8 (26.1–27.6)	26.5 (25.7–27.2)	
Smoker	12.6 (12.2–13.0)	15.9 (15.3–16.6)	9.6 (9.2–10.2)	
Alcohol abuse				<0.001
No	82.9 (82.4–83.4)	74.0 (73.2–74.8)	90.8 (90.3–91.3)	
Yes	17.1 (16.6–17.6)	26.0 (25.2–26.8)	9.2 (8.7–9.7)	
Leisure-time physical activity				
Active	30.1 (29.4–30.7)	34.2 (33.3–35.1)	26.4 (25.6–27.1)	<0.001
Inactive	69.9 (69.3–70.6)	65.8 (64.9–66.7)	73.6 (72.9–74.4)	
Recommended fruit and vegetable consumption				
No	87.0 (86.6–87.5)	89.8 (89.2–90.3)	84.6 (84.0–85.2)	<0.001
Yes	13.0 (12.5–13.4)	10.2 (9.7–10.8)	15.4 (14.8–16.0)	
Ultra-processed food consumption				
No	85.7 (85.2–86.2)	84.3 (83.6–85.0)	86.9 (86.3–87.5)	<0.001
Yes	14.3 (13.8–14.8)	15.7 (15.0–16.4)	13.1 (12.5–13.7)	
Regular consumption of soft drinks and/or artificial juices				
No	67.1 (66.5–67.7)	62.8 (61.9–63.6)	70.9 (70.2–71.7)	<0.001
Yes	32.9 (32.3–33.5)	37.2 (36.4–38.1)	29.1 (28.3–29.8)	
Nutritional status				
Low weight	2.2 (2.0–2.4)	1.6 (1.40–1.9)	2.7 (2.4–2.9)	<0.001
Normal weight	39.9 (39.3–40.5)	39.1 (38.3–40)	40.5 (39.7–41.3)	
Overweight	36.7 (36.1–37.4)	40.0 (39.1–40.9)	33.8 (33.0–34.6)	
Obese	21.2 (20.6–22.0)	19.2 (18.4–20.1)	23.1 (22.3–23.9)	

National Health Survey, Brazil, 2019. 95% CI, 95% Confidence Interval.

^*^Missing data: 9 (6 men and 3 women).

^†^The prevalence values are weighted by the complex sample.

^§^Chi-square test adjusted by study design.

The average number of diseases in the study population was 1.13 (standard deviation = 0.01; 95% CI: 1.11–1.15; minimum 0 and maximum 12) and was lower in men (0.88, standard deviation = 0.01; 95% CI: 0.86–0.90; minimum 0 and maximum 10) compared to women (1.34, standard deviation = 1.34; 95% CI: 1.31–1.37; minimum 0 and maximum 12) (*p* < 0.001).

### 3.2. Prevalence of multimorbidity

Multimorbidity prevalence was 29.4% (95% CI: 28.9–30.0), being higher among women (35.4%; 95% CI: 34.6–36.2) compared to men (22.7%; 95% CI: 21.9–23.4) (*p* < 0.001). Prevalence rates of multimorbidity also increased substantially with age for the total sample and both sexes (*p* < 0.001). Compared to 8.3% (95% CI: 7.3–9.4) of people aged 18–24 years, the prevalence of multimorbidity in the 40–59 age group (34.1%; 95% CI: 33.2–35.1) and ≥ 60 years (56.5%; 95% CI: 55.4–57.6) increased significantly. Regardless of sex, the most reported morbidities were hypertension (23.9%), chronic back problems (21.6%), and hypercholesterolemia (14.6%; Table 2).

**Table 2:** Prevalence of morbidities and multimorbidity in the study population, according to sex and age group.

Morbidities	Age group (years)					
	%^†^ (95% CI)	%^†^ (95% CI)	%^†^ (95% CI)	%^†^ (95% CI)	%^†^ (95% CI)	*p*-value^§^
Total	Total (*n* = 88,531)	18–24 (*n* = 8,145)	25–39 (*n* = 25,399)	40–59 (*n* = 32,259)	≥60 (*n* = 22,728)	
Diabetes	7.7 (7.4–8.0)	0.7 (0.5–1.1)	1.6 (1.3–1.9)	7.9 (7.4–8.5)	20.2 (19.3–21.1)	<0.001
Hypertension	23.9 (23.4–24.4)	2.3 (1.8–2.9)	7.2 (6.7–7.8)	27.2 (26.3–28.8)	55.0 (53.9–56.1)	<0.001
Stroke	2.0 (1.8–2.1)	0.3 (0.1–1.0)	0.3 (0.2–0.4)	1.7 (1.5–2.0)	5.6 (5.1–6.1)	<0.001
Chronic heart disease	5.3 (5.0–5.6)	1.4 (0.9–2.2)	2.0 (1.6–2.3)	4.8 (4.4–5.2)	13.1 (12.4–13.9)	<0.001
Arthritis/rheumatism	7.6 (7.2–7.9)	0.7 (0.5–1.1)	2.0 (1.7–2.3)	8.4 (7.7–9.1)	18.2 (17.2–19.1)	<0.001
Chronic lung disease^*^	1.7 (1.5–1.8)	1.2 (0.8–1.7)	1.1 (0.9–1.4)	1.5 (1.3–1.9)	2.9 (2.5–3.4)	<0.001
Asthma	5.3 (5.0–5.6)	7.3 (6.3–8.4)	5.3 (4.8–5.8)	4.9 (4.5–5.4)	4.6 (4.1–5.1)	<0.001
Chronic kidney failure	1.5 (1.3–1.6)	0.8 (0.5–1.2)	0.8 (0.6–1.0)	1.6 (1.4–1.9)	2.6 (2.2–2.9)	<0.001
Cancer	2.6 (2.4–2.7)	0.1 (0.1–0.3)	0.7 (0.6–0.9)	2.4 (2.2–2.8)	6.8 (6.2–7.4)	<0.001
Hypercholesterolemia	14.6 (14.1–15.0)	3.5 (2.8–4.2)	6.7 (6.1–7.3)	17.7 (17.0–18.5)	27.2 (26.2–28.3)	<0.001
WR-MSD	2.5 (2.3–2.8)	0.6 (0.3–1.0)	2.0 (1.7–2.4)	3.9 (3.4–4.4)	2.2 (1.8–2.7)	<0.001
Chronic back problem	21.6 (21.0–22.1)	9.3 (8.3–10.5)	14.6 (13.8–15.4)	26.4 (25.5–27.3)	31.1 (30–32.2)	<0.001
Depression	10.2 (9.9–10.6)	5.9 (4.9–7.0)	8.1 (7.5–8.7)	12.7 (12.1–13.4)	11.8 (11.1–12.6)	<0.001
Mental disorders	6.5 (6.1–6.8)	6.9 (5.9–8.0)	7.0 (6.4–7.5)	7.0 (6.5–7.5)	4.7 (4.2–5.2)	<0.001
Multimorbidity	29.4 (28.9–30.0)	8.3 (7.3–9.4)	13.8 (13.1–14.6)	34.1 (33.2–35.1)	56.5 (55.4–57.6)	<0.001
Men	Total (*n* = 41,662)	18–24 (*n* = 3,864)	25–39 (*n* = 12,253)	40–59 (*n* = 15,352)	≥60 (*n* = 10,193)	
Diabetes	6.9 (6.5–7.4)	1.0 (0.6–1.9)	1.3 (0.9–1.7)	7.4 (6.7–8.2)	18.9 (17.6–20.3)	<0.001
Hypertension	21.1 (20.4–21.8)	2.5 (1.7–3.6)	7.4 (6.6–8.2)	24.6 (23.4–25.9)	49.3 (47.6–50.9)	<0.001
Stroke	2.0 (1.7–2.2)	0.1 (0.0–0.1)	0.2 (0.1–0.3)	1.7 (1.4–2.1)	6.4 (5.6–7.3)	<0.001
Chronic heart disease	4.9 (4.6–5.3)	1.6 (0.8–3.2)	1.7 (1.2–2.3)	4.5 (3.9–5.1)	13.2 (12.1–14.3)	<0.001
Arthritis/rheumatism	3.7 (3.4–4.1)	0.2 (0.1–0.4)	1.4 (1.0–1.8)	4.6 (3.8–5.6)	8.3 (7.5–9.2)	<0.001
Chronic lung disease^*^	1.6 (1.4–1.8)	1.2 (0.7–2.0)	1.0 (0.8–1.4)	1.4 (1.1–1.7)	3.0 (2.5–3.6)	<0.001
Asthma	4.4 (4.0–4.8)	7.4 (6.0–9.2)	4.6 (4.0–5.2)	3.4 (2.9–4.0)	3.5 (3.0–4.2)	<0.001
Chronic kidney failure	1.4 (1.2–1.6)	0.9 (0.5–1.8)	0.8 (0.6–1.2)	1.3 (1.0–1.6)	2.7 (2.2–3.3)	<0.001
Cancer	2.1 (1.9–2.4)	0.2 (0.0–0.5)	0.4 (0.2–0.5)	1.5 (1.2–1.8)	7.4 (6.6–8.4)	<0.001
Hypercholesterolemia	11.1 (10.6–11.7)	2.9 (2.1–4.0)	5.5 (4.8–6.3)	14.3 (13.3–15.4)	19.9 (18.6–21.3)	<0.001
WR-MSD	1.7 (1.5–2.0)	0.7 (0.3–1.5)	1.4 (1.1–1.9)	2.7 (2.2–3.2)	1.4 (1.0–1.8)	<0.001
Chronic back problem	18.3 (17.6–19.0)	7.8 (6.5–9.4)	12.7 (11.7–13.8)	2.35 (22.3–24.7)	25.5 (24.2–26.9)	<0.001
Depression	5.1 (4.7–5.5)	3.8 (2.7–5.4)	4.0 (3.4–4.7)	5.8 (5.2–6.4)	6.6 (5.8–7.5)	<0.001
Mental disorders	4.1 (3.8–4.5)	4.9 (3.8–6.3)	4.3 (3.7–5.0)	4.4 (3.9–5.0)	2.6 (2.2–3.2)	<0.001
Multimorbidity	22.7 (21.9–23.4)	6.4 (5.1–8.0)	10.0 (9.0–11.0)	26.3 (25.1–27.6)	47.5 (45.9–49.0)	<0.001
Women	Total (*n* = 46,869)	18–24 (*n* = 4,281)	25–39 (*n* = 13,146)	40–59 (*n* = 16,907)	≥60 (*n* = 12,535)	
Diabetes	8.4 (8.0–8.9)	0.4 (0.3–0.7)	1.8 (1.5–2.3)	8.4 (7.7–9.2)	21.2 (20.0–22.4)	<0.001
Hypertension	26.4 (25.8–27.2)	2.1 (1.5–2.9)	7.2 (6.4–8.0)	29.5 (28.2–30.7)	59.4 (57.9–60.8)	<0.001
Stroke	2.0 (1.8–2.2)	0.6 (0.2–1.9)	0.4 (0.3–0.6)	1.7 (1.4–2.1)	5.0 (4.4–5.6)	<0.001
Chronic heart disease	5.6 (5.3–6.0)	1.2 (0.8–1.7)	2.2 (1.8–2.7)	5.1 (4.6–5.7)	13.1 (12.1–14.2)	<0.001
Arthritis/rheumatism	11.0 (10.4–11.5)	1.2 (0.8–1.9)	2.6 (2.1–3.1)	11.6 (10.8–12.6)	25.7 (24.3–27.2)	<0.001
Chronic lung disease^*^	1.7 (1.5–2.0)	1.2 (0.7–1.9)	1.2 (0.9–1.6)	1.7 (1.3–2.3)	2.8 (2.3–3.5)	<0.001
Asthma	6.1 (5.7–6.5)	7.1 (5.9–8.5)	6.0 (5.3–6.7)	6.2 (5.5–6.9)	5.3 (4.7–6.1)	0.075
Chronic kidney failure	1.6 (1.4–1.8)	0.7 (0.4–1.1)	0.7 (0.5–1.0)	1.9 (1.6–2.4)	2.4 (2.0–3.0)	<0.001
Cancer	2.9 (2.7–3.2)	0.1 (0.0–0.3)	1.1 (0.8–1.4)	3.3 (2.8–3.8)	6.3 (5.6–7.1)	<0.001
Hypercholesterolemia	17.6 (17.0–18.3)	4.0 (3.1–5.1)	7.8 (7.0–8.7)	20.6 (19.6–21.7)	32.8 (31.4–34.3)	<0.001
WR-MSD	3.2 (2.8–3.6)	0.5 (0.3–0.8)	2.5 (2.0–3.1)	4.9 (4.2–5.7)	2.9 (2.3–3.6)	<0.001
Chronic back problem	24.5 (23.7–25.2)	10.9 (9.4–12.5)	16.3 (15.2–17.5)	28.9 (27.7–30.2)	35.3 (33.8–36.8)	<0.001
Depression	14.7 (14.1–15.4)	8.0 (6.6–9.6)	12.0 (11.0–13.0)	18.8 (17.7–19.9)	15.8 (14.7–17.0)	<0.001
Mental disorders	8.6 (8.1–9.1)	8.9 (7.4–10.8)	9.4 (8.6–10.4)	9.2 (8.5–10.0)	6.2 (5.5–7.0)	<0.001
Multimorbidity	35.4 (34.6–36.2)	10.2 (8.8–11.9)	17.4 (16.3–18.6)	40.9 (39.7–42.2)	63.4 (61.9–64.8)	<0.001

National Health Survey, Brazil, 2019. WR-MSD, Work-related musculoskeletal disorder; 95% CI, 95% Confidence Interval.

^*^Pulmonary emphysema, chronic bronchitis, or chronic obstructive pulmonary disease (COPD).

^†^The prevalence values are weighted by the complex sample.

^§^Chi-square test adjusted by study design.

The supplementary tables display the results of the bivariate Poisson regression analysis in the total sample (Supplementary Table S3), men (Supplementary Table S4), and women (Supplementary Table S5).

### 3.3. Factors associated with multimorbidity in the total sample

After adjusting the multiple regression model, a positive gradient in the multimorbidity prevalence with age was observed in the study population, with the greatest magnitude for the older people (APR: 6.25; 95% CI: 4.75–8.23). The prevalence of multimorbidity was higher among women (APR: 1.47, 95% CI: 1.42–1.53), residents of the South (APR: 1.99, 95% CI: 1.34–2.96) and Southeast (APR: 1.58, 95% CI: 1.07–2.33) regions of Brazil, urban residents (APR: 1.10, 95% CI: 1.06–1.15), former smokers (APR: 1.24, 95% CI: 1.20–1.29), current smokers (APR: 1.09, 95% CI: 1.04–1.16), physically inactive (APR: 1.07, 95% CI: 1.03–1.11), overweight (APR: 1.24, 95% CI: 1.20–1.29), and obese (APR: 1.52, 95% CI: 1.46–1.59) individuals. In turn, a lower prevalence was found for participants with higher educational level (complete high school/incomplete higher education; *p* = 0.002) and for people who reported insufficient fruit and vegetable consumption (*p* = 0.003) (Table 3). The results of the interaction terms exhibited significant interactions between age group/geographic region, education/self-declared race/skin color, and geographic region/self-declared race/skin color (Supplementary Table S4).

**Table 3:** Multiple regression analysis of factors associated with multimorbidity in the total sample.

Variables	APR	95% CI	*p*-value^||^
Sex			
Male	1.00		
Female	1.47	1.42–1.53	<0.001
Age group (years)			
18–24	1.00		
25–39	1.71	1.27–2.30	<0.001
40–59	3.84	2.92–5.05	<0.001
≥60	6.25	4.75–8.23	<0.001
Self-declared race/skin color			
White	1.06	0.92–1.22	0.406
Brown	1.00		
Black	0.90	0.71–1.14	0.373
Others (yellow or indigenous)	0.82	0.54–1.25	0.355
Education			
No education or incomplete middle school	0.98	0.86–1.13	0.814
Complete middle school or incomplete high school	0.83	0.69–1.01	0.056
Complete high school or incomplete higher education	0.87	0.67–0.91	0.002
Complete higher education or more	1.00		
Geographic region			
North	1.00		
Northeast	1.14	0.78–1.67	0.488
Southeast	1.58	1.07–2.33	0.022
South	1.99	1.34–2.96	0.001
Midwest	1.49	0.94–2.38	0.092
Area of residence			
Urban	1.10	1.06–1.15	<0.001
Rural	1.00		
Tobacco use			
Non-smoker	1.00		
Former smoker	1.24	1.20–1.29	<0.001
Smoker	1.09	1.04–1.16	0.001
Alcohol abuse			
No	1.00		
Yes	0.96	0.90–1.01	0.116
Leisure-time physical activity			
Active	1.00		
Inactive	1.07	1.03–1.11	0.001
Recommended fruit and vegetable consumption			
No	0.94	0.89–0.98	0.003
Yes	1.00		
Ultra-processed food consumption			
No	1.00		
Yes	1.02	0.89–1.17	0.79
Regular consumption of soft drinks and/or artificial juices			
No	1.00		
Yes	0.97	0.93–1.01	0.178
Nutritional status			
Low weight	1.03	0.89–1.20	0.645
Normal weight	1.00		
Overweight	1.24	1.20–1.29	<0.001
Obese	1.52	1.46–1.59	<0.001

National Health Survey, Brazil, 2019. 95% CI, 95% Confidence Interval; APR, Adjusted Prevalence Ratio.

^**||**^Wald chi-square test.

### 3.4. Factors associated with multimorbidity according to sex

Similar associations were found between men (Table 4) and women (Table 5). However, we observed that some associations differed between sexes, where people aged 25–39 years (*p* < 0.001), residents of the South (*p* < 0.001) and Midwest (*p* = 0.039) regions of Brazil were associated with a higher prevalence of multimorbidity only in women. Physical inactivity was positively associated with a higher prevalence of multimorbidity only in men (*p* = 0.001). The educational levels complete middle school/incomplete high school (*p* = 0.031) and complete high school/incomplete higher education (*p* < 0.001) were associated with a lower prevalence of multimorbidity only in men. The results of the interaction terms in men showed significant interactions between education/self-reported race/skin color and geographic region/self-reported race/skin color (Supplementary Table S5). In women, significant interactions were observed between age group/geographic region, education/self-reported race/skin color, geographic region/self-reported race/skin color, and ultra-processed food consumption/recommended fruit and vegetable consumption (Supplementary Table S6).

**Table 4:** Multiple regression analysis of factors associated with multimorbidity in men.

Variables	APR	95% CI	*p*-value^||^
Age group (years)			
18–24	1.00		
25–39	1.44	0.90–2.32	0.131
40–59	3.29	2.12–5.11	<0.001
≥60	6.05	3.94–9.30	<0.001
Self-declared race/skin color			
White	1.25	0.98–1.59	0.067
Brown	1.00		
Black	0.96	0.65–1.42	0.855
Others (yellow or indigenous)	0.79	0.36–1.76	0.567
Education			
No education or incomplete middle school	0.85	0.65–1.11	0.231
Complete middle school or incomplete high school	0.69	0.49–0.97	0.031
Complete high school or incomplete higher education	0.63	0.46–0.85	<0.001
Complete higher education or more	1.00		
Geographic region			
North	1.00		
Northeast	1.31	0.68–2.52	0.415
Southeast	1.59	0.83–3.02	0.161
South	1.75	0.90–3.40	0.100
Midwest	1.08	0.45–2.64	0.857
Area of residence			
Urban	1.17	1.04–1.20	<0.001
Rural	1.00		
Tobacco use			
Non-smoker	1.00		
Former smoker	1.34	1.26–1.43	<0.001
Smoker	1.12	1.02–1.23	0.023
Alcohol abuse			
No	1.00		
Yes	0.83	0.65–1.06	0.137
Leisure-time physical activity			
Active	1.00		
Inactive	1.12	1.04–1.20	0.001
Recommended fruit and vegetable consumption			
No	0.90	0.82–0.99	0.035
Yes	1.00		
Ultra-processed food consumption			
No	1.00		
Yes	0.83	0.65–1.06	0.137
Regular consumption of soft drinks and/or artificial juices			
No	1.00		
Yes	0.98	0.91–1.05	0.178
Nutritional status			
Low weight	0.98	0.75–1.28	0.895
Normal weight	1.00		
Overweight	1.29	1.20–1.38	<0.001
Obese	1.62	1.50–1.76	<0.001

National Health Survey, Brazil, 2019. 95% CI, 95% Confidence Interval; APR, Adjusted Prevalence Ratio.

^**||**^Wald chi-square test.

**Table 5:** Multiple regression analysis of factors associated with multimorbidity in women.

Variables	APR	95% CI	*p*-value^||^
Age group (years)			
18–24	1.00		
25–39	1.87	1.30–2.70	<0.001
40–59	4.16	2.96–8.85	<0.001
≥60	6.24	4.43–8.81	<0.001
Self-declared race/skin color			
White	0.96	0.81–1.14	0.639
Brown	1.00		
Black	0.90	0.68–1.21	0.492
Others (yellow or indigenous)	0.85	0.56–1.31	0.467
Education			
No education or incomplete middle school	1.08	0.92–1.27	0.323
Complete middle school or incomplete high school	0.93	0.75–1.15	0.523
Complete high school or incomplete higher education	0.88	0.73–1.05	0.156
Complete higher education or more	1.00		
Geographic region			
North	1.00		
Northeast	1.07	0.68–1.68	0.762
Southeast	1.57	0.97–2.53	0.066
South	2.16	1.33–3.52	<0.001
Midwest	1.78	1.03–3.08	0.039
Area of residence			
Urban	1.06	1.01–1.11	0.026
Rural	1.00		
Tobacco use			
Non-smoker	1.00		
Former smoker	1.18	1.13–1.23	<0.001
Smoker	1.09	1.02–1.17	0.009
Alcohol abuse			
No	1.00		
Yes	0.99	0.91–1.09	0.876
Leisure-time physical activity			
Active	1.00		
Inactive	1.05	0.99–1.10	0.083
Recommended fruit and vegetable consumption			
No	0.94	0.90–0.99	0.018
Yes	1.00		
Ultra-processed food consumption			
No	1.00		
Yes	1.11	0.95–1.29	0.194
Regular consumption of soft drinks and/or artificial juices			
No	1.00		
Yes	0.97	0.92–1.02	0.259
Nutritional status			
Low weight	1.04	0.88–1.24	0.639
Normal weight	1.00		
Overweight	1.22	1.16–1.28	<0.001
Obese	1.46	1.39–1.54	<0.001

National Health Survey, Brazil, 2019. 95% CI, 95% Confidence Interval; APR, Adjusted Prevalence Ratio.

^**||**^Wald chi-square test.

## 4. Discussion

Using a nationally representative sample, we found that multimorbidity was prevalent in the Brazilian adult population. Alarmingly, one in four Brazilian adults had multimorbidity. Overall, multimorbidity was more prevalent among women, the older people, residents of the South and Southeast regions, urban area residents, former smokers, current smokers, physically inactive, overweight, and obese individuals. In turn, individuals with complete high school/incomplete higher education showed a lower prevalence of multimorbidity than those with higher education. The associations between education and multimorbidity differed between sexes. In men, multimorbidity was inversely associated with the strata of complete middle school/incomplete high school and complete high school/incomplete higher education, while in women, the association was not significant. The South and Midwest regions were associated with a higher prevalence in women, while leisure-time physical inactivity remained associated only in men. The recommended fruit and vegetable consumption was inversely related to multimorbidity for the total sample and both sexes.

The prevalence found in this study was similar to that estimated in low- and middle-income countries (29.7%), according to a meta-analysis involving 1,180,111 individuals from 57 countries (4). The prevalence ratio of multimorbidity is higher in high-income countries compared to low- and middle-income countries, possibly due to differences in diagnostic and data management systems between countries (4).

The results were also higher than those found in the Brazilian adult population in 2013 (23.6%), indicating an increase in multimorbidity burden in Brazil (43). This increment was reported in different countries (44, 45). The rise in multimorbidity is related to demographic, nutritional, and epidemiological transition, besides other determinants such as urbanization (46).

Regardless of sex, we observed increasing multimorbidity with age. This association is well described in the literature (3, 4). Different mechanisms may explain this relationship. First, due to the demographic transition that is responsible for the increase in the number of older people (47). Second, changes inherent to aging predispose to the development of chronic diseases/conditions (48). Several physiological changes, for example, are associated with senescence, such as increased body fat, especially in women, which often leads to a higher prevalence of overweight and obesity; decrease in fat-free mass and reduced capacity of the glucose uptake system, contributing to insulin resistance and diabetes; and reduced cardiac output, which can lead to increased cardiovascular disease risk (49). Other mechanisms have also been associated with a higher incidence of chronic diseases/conditions (49). Third, owing to the accumulation of risk factors. Longevity is related to longer exposure to these covariates, such as smoking status, unhealthy diet, alcohol consumption, and physical inactivity (50). Therefore, as the older people population grows, an increasing prevalence of multimorbidity is expected, with a potential overload of health care systems (12). Despite this, our results indicated that multimorbidity is not restricted to elderly. Indeed, younger people showed a high prevalence, consistent with previous observations (19, 23, 43).

Women had a higher prevalence of multimorbidity than men, corroborating other studies (4, 16, 51). One of the most consistent explanations for this result is a possible residual confounding due to higher consultation rates in women, leading to higher rates of chronic disease/condition diagnosis, regardless of age (52). In fact, in this investigation, women had a higher prevalence of multimorbidity than men in all age strata. Another explanation would be related to survival bias, as men have a lower life expectancy (53). Thus, the burden of chronic diseases/conditions in women tends to increase.

The lowest prevalence of multimorbidity was observed among participants with complete high school/incomplete higher education compared to people with higher education in the study population, which is in agreement with previous findings (54–56). A meta-analysis reported that low education level was associated with a 64% increased odds of multimorbidity (11). Considering only low- and middle-income countries, such as Brazil, earlier studies have shown inconsistencies, with a positive association (19, 57, 58), negative association (32, 59), or no significant difference between education and multimorbidity (60). Behavioral aspects are probably related to this result. The prevalence of risk factors differs by educational level; smoking, unhealthy eating habits, and obesity are more frequent among less educated individuals. In turn, leisure-time physical activity and the recommended fruit and vegetable consumption are higher among people with higher education (34). For example, a study carried out with Chinese residents showed that participants with a high level of education had positive knowledge, attitudes and behaviors related to diet that contributed to the prevention of hypertension (61).

Our findings suggest different associations between education and multimorbidity by sex. Intermediate educational levels (complete middle school/incomplete high school and complete high school/incomplete higher education) were a protective factor for multimorbidity among men, while this association was not verified among women. Previous investigations demonstrated relevant similarities (55, 62). However, a systematic review reported that most works found associations for sex-matched samples, thereby limiting the scope of evidence stratified by sex (11). The relationship between education and multimorbidity by sex can be explained, besides other factors, by differences in the magnitude between low education and the most prevalent chronic diseases in the study (55). For example, additional analysis showed that the strength of the association between low education (no education/incomplete middle education) and diseases, such as diabetes (PR_women_: 4.22 versus PR_men_: 1.69), hypertension (PR_women_: 2.65 versus PR_men_: 1.41), and high cholesterol (PR_women_: 1.73 versus PR_men_: 0.77), is higher in women compared to men (data not shown in the results).

We found a higher prevalence of multimorbidity among urban residents compared to rural residents, regardless of sex, which is in accordance with previous reports (51, 63, 64). People in urban areas have different behavior patterns and lifestyles than those in rural areas. Urban residents, for example, have a higher consumption of energy-dense foods, rich in sugar, trans fats, and sodium (65), as they are more exposed to ultra-processed foods and marketing strategies of food companies (66). These ultra-processed foods require less preparation time, hence meeting the needs of urban populations who work outside the home and may have limited time or resources to cook (67). Furthermore, scientific literature reports that urban residents spend less time on moderate and vigorous-intensity physical activity, and consume more alcohol, increasing the likelihood of developing multiple chronic diseases/conditions (68).

The five regions of Brazil showed inequalities in the prevalence of multimorbidity. The South and Southeast regions had higher prevalence, similar to that previously reported (63, 69, 70). No significant associations were observed in men. Conversely, women living in the South and Midwest regions had a higher multimorbidity prevalence than the others, corroborating a study conducted with a similar population (71). These outcomes probably reflect socioeconomic disparities in access to health services between sexes and regions. For example, states in the South and Southeast regions, which include the cities of São Paulo and Rio de Janeiro, have a higher percentage of urban population (72), higher level of socioeconomic development (73), are more industrialized, with better infrastructure (74) and have greater access to health services (75) compared to the North and Northeast regions. These aspects can facilitate access to the diagnosis of chronic diseases/conditions (76). In Brazil, the North (1.0/1,000 inhabitants) and Northeast (1.2/1,000 inhabitants) regions have the lowest ratio of doctors per 1,000 inhabitants in comparison to the Southeast (2.6/1,000 inhabitants) and South (2.0/1,000 inhabitants) (76). Another explanation for this result is the difference in demographic and epidemiological transition processes between the Brazilian regions. Data from the Global Burden of Disease Study 2016 showed that the transition from communicable, maternal, neonatal, and nutritional diseases to NCDs is more advanced in the South and Southeast regions, in addition to the higher life expectancy in these regions compared to the others (77).

Our findings revealed that smoking (former and current smokers) was positively associated with a higher prevalence of multimorbidity, regardless of sex, confirming existing literature (13–15). The greatest magnitude of association was observed in former smokers, which is in line with other investigations (78, 79). The cross-sectional nature of this study does not allow to establish causality between smoking cessation and multimorbidity. However, longitudinal studies have shown that a former history of smoking and current smoking increase the risk of multimorbidity (80, 81). Smoking is associated with the occurrence of multiple diseases. Recent research indicated that smoking was associated with 470 diseases, such as lung cancer, COPD, acute myocardial infarction, ischemic stroke, and ischemic heart disease (82). Cigarette smoke contains more than 7,000 chemical substances, of which approximately 70 are carcinogens. For instance, inhaling these chemicals leads to changes in airway inflammation, resulting in abnormal processes that can cause serious smoking-related illnesses, such as cancer and COPD (83, 84).

We observed that leisure-time physical inactivity was associated with multimorbidity in the total sample, as found in other studies (13–15). In the analysis stratified by sex, this association was verified only in men, corroborating previous investigations (85, 86). Another research estimated that physical inactivity is a primary cause of 35 chronic diseases/conditions that affect different body systems (87). Physical activity confers benefits for several health outcomes, such as improved physical fitness (cardiorespiratory and muscular fitness), cardiometabolic health (blood pressure, dyslipidemia, and insulin resistance), bone health, mental health, and reduced adiposity (88).

We found an inverse association between recommended fruit and vegetable consumption and multimorbidity, regardless of sex. Evidence suggests that high consumption of fruits and vegetables is a protective factor for several NCDs, such as obesity (89), ischemic stroke (90), and diabetes (91), contrary to our findings. This can be explained by the following factors: (i) information on the recommended fruit and vegetable consumption is self-reported in the NHS. Although this indicator provided satisfactory validity (92–94), response bias may have occurred; also, the data refer to the day before the interview, not reflecting the relationship between lifetime consumption and multimorbidity; (ii) people with multimorbidity tend to adopt healthier eating habits (17); and (iii) additional analysis (not shown in the results) demonstrated that the recommended fruit and vegetable consumption was twice as high in the older people compared to young people who, in turn, had a prevalence of multimorbidity almost seven times lower. This inverse association between healthy eating indicators and age may reflect a greater concern with health among older people, as described in the literature (20, 65, 95–98). Additionally, given the disagreements about the positive relationship between multimorbidity and recommended fruit and vegetable consumption (15, 16, 18, 19, 99), further in-depth investigations are required, especially using Food-Frequency Questionnaires. However, studies using data from the NHS 2013 (17, 18) showed similar results to ours.

The associations between overweight and multimorbidity are well known (18, 81, 100, 101), as found in this work. Obesity can lead to dyslipidemia, systemic inflammation, increased blood pressure, insulin resistance, and other metabolic changes, which may be common pathways for the development of cardiovascular disease and diabetes (102).

The results of this study indicate that the low educational level of the population and physical inactivity are determining factors for multimorbidity. Emphasizing the importance of actions that reduce health inequalities and promote the practice of physical activity. In addition, we showed that the older adults and women are vulnerable groups for the occurrence of multimorbidity. Therefore, tailored interventions for these groups are needed to prevent a scenario with an even higher prevalence of multimorbidity and its burden on the health system in the future.

This study has some limitations. First, the cross-sectional nature does not allow to establish causality between the independent variables and multimorbidity, as there may be reverse causality. Thus, longitudinal studies are needed. Second, data on chronic diseases/conditions were self-reported, considered less accurate than objective measures, and susceptible to memory and response bias. Therefore, some morbidities may be underestimated. Third, the definition of multimorbidity was based on the number of chronic diseases/conditions without considering severity. Ultimately, the analysis was based on a list of only 14 morbidities and the interviewee’s report of a medical diagnosis, which has a strong relationship with access to health services and may reduce the frequency of multimorbidity among the socioeconomically disadvantaged population. Moreover, mental disorders were grouped, and anxiety, the most prevalent mental illness in Brazil (103), was not analyzed. However, our investigation has several strengths, such as the complex sampling design, which makes it representative of the Brazilian adult population. Also, we examined the association of multimorbidity with sociodemographic and lifestyle factors stratified by sex. The results are valuable for future evidence-based recommendations, such as gender-specific prevention strategies to address multimorbidity. Importantly, our findings can be used to provide a deeper understanding of the association between sociodemographic and lifestyle determinants and multimorbidity in developing countries, considering the knowledge gap in the literature.

In conclusion, the higher prevalence of multimorbidity was associated with sociodemographic characteristics, including older age, female sex, living in the South and Southeast regions of Brazil, living in urban areas, in addition to unhealthy lifestyles, such as smoking and physical inactivity. Overweight and obesity also increased the prevalence of multimorbidity. Intriguingly, the recommended fruit and vegetable consumption demonstrated an inverse association. Compared with participants with higher educational levels, those with intermediate education (complete high school/incomplete higher education) had a lower prevalence of multimorbidity. In men and women, the association between education and multimorbidity showed differences, as well as for physical activity.

## Data availability statement

Publicly available datasets were analyzed in this study. This data can be found: Brazilian Institute of Geography and Statistics with the Ministry of Health/Brazil (https://biblioteca.ibge.gov.br/index.php/biblioteca-catalogo?view=detalhes&id=2101764).

## Ethics statement

The studies involving human participants were reviewed and approved by the Research Ethics Committee of the National Health Council under protocol number 3,529,376/2019. The patients/participants provided their written informed consent to participate in this study.

## Author contributions

RG contributed to the conception and design of the study. CCP and RG organized the database and performed the statistical analysis. CCP wrote the first draft of the manuscript. CFP and SB wrote sections of the manuscript. All authors contributed to manuscript revision, read, and approved the submitted version.

## Funding

This study was funded by the Ministry of Health of Brazil.

## Conflict of interest

The authors declare that the research was conducted in the absence of any commercial or financial relationships that could be construed as a potential conflict of interest.

## Publisher’s note

All claims expressed in this article are solely those of the authors and do not necessarily represent those of their affiliated organizations, or those of the publisher, the editors and the reviewers. Any product that may be evaluated in this article, or claim that may be made by its manufacturer, is not guaranteed or endorsed by the publisher.
