# Supplementary material for: Prevalence and factors associated with multimorbidity in adults in Brazil, according to sex: a population-based cross-sectional survey
Source: Front Public Health. 2023 Jun 2;11:1193428. doi: 10.3389/fpubh.2023.1193428 (PMC10278573; doi:10.3389/fpubh.2023.1193428)
Supplement: Supplementary file 1 [file Table_1.docx]

Supplementary Material

Prevalence and factors associated with multimorbidity in adults in Brazil, according to sex: a population-based cross-sectional survey

**Cristina Camargo Pereira, Charlise Fortunato Pedroso, Sandro Rogério Rodrigues Batista, Rafael Alves Guimarães^*^**

*** Correspondence:** Rafael Alves Guimarães: [rafaelalves@ufg.br](mailto:rafaelalves@ufg.br)

**Supplementary Table 1.** List of the 14 disease conditions included in multimorbidity count

| **Morbidities** | **Questions** |
| --- | --- |
| Hypertension | *“Has a doctor ever diagnosed you with arterial hypertension (high blood pressure)?”* |
| Diabetes | *“Has a doctor ever diagnosed you with diabetes?”* |
| Hypercholesterolemia | *“Has a doctor ever diagnosed you with high cholesterol?”* |
| Chronic heart disease | *“Has a doctor ever diagnosed you with a heart disease, such as a heart attack, angina, heart failure or something else?”* |
| Stroke | *“Has a doctor ever diagnosed you with a CVA (Cerebral Vascular Accident) or stroke?”* |
| Asthma | *“Has a doctor ever diagnosed you with asthma (or asthmatic bronchitis)?”* |
| Arthritis/rheumatism | *“Has a doctor ever diagnosed you with arthritis or rheumatism?”* |
| Chronic back problem | *“Do you have any chronic back problem, such as chronic back or neck pain, low back pain, sciatica, vertebrae, or disc problems?”* |
| Work-related musculoskeletal disorder | *“Has a doctor ever diagnosed you with Work-related musculoskeletal disorder (WR-MSD)?”* |
| Depression | *“Has a doctor or mental health professional (psychiatrist or psychologist) diagnosed you with depression?”* |
| Mental disorders | *“Has a doctor or health professional (psychiatrist or psychologist) diagnosed you with another mental illness such as anxiety disorder, panic disorder, schizophrenia, bipolar disorder, psychosis, or OCD (obsessive-compulsive disorder)?”* |
| Chronic lung disease | *“Has a doctor ever diagnosed you with chronic lung disease such as pulmonary emphysema, chronic bronchitis or chronic obstructive pulmonary disease - COP)?”* |
| Cancer | *“Has a doctor ever diagnosed you with cancer?”* |
| Chronic kidney failure | *“Has a doctor ever diagnosed you with Chronic kidney failure?”* |

**Supplementary Table 2.** Independent variables of the study

| **Variables** | **Questions** | **Answer options** | **Operacional definitions** | **Analysis categories** |
| --- | --- | --- | --- | --- |
| Sex | *Sex* | *“Male” or “Female”.* | - | “Male” or “Female” |
| Age group | *Resident's age on the reference date* | Age in years. | The division of the sample according to age group into four groups: young people, young adult, adults and older adults (60). | “18-24 years”; “25-39 years”; “40-59 years”; or “≥60 years”. |
| Self-declared race/skin color | *Color or race:* | *“white”; “black”; “brown”; “yellow” or “indigenous”.* | Self-declaration (27). | “White”; “Black”; “Brown”; or “Others (yellow or indigenous)”. |
| Education | *“Did you previously attend school or daycare? (School included pre-school through PhD)”* | *“Yes” or “No”.* | Education level (28). | “No education/incomplete middle school”; “Complete middle school/incomplete high school”; “Complete high school/incomplete higher education”; or “Complete higher education or more”. |
|  | *“What was the highest course you attended?”* | *“Nursery”; “Preschool”; “Literacy class – CA”; “Literacy for youth and adults”; “Former primary (elementary)”; “Former high school (1st cycle high school)”; “Regular elementary school or 1st grade”; “Youth and adult education (EJA) or supplementary elementary education”; “Ancient scientific; classic etc. (medium 2nd cycle)”; “Regular high school or high school”; “Youth and adult education (EJA) or supplementary secondary education”; “Higher – graduation”; “Higher level specialization (minimum duration of 360 hours)”; “Master's degree”; “Doctorate degree”; “Ignored”; or “Not applicable”.* |  |  |
| Living with a spouse/partner | *“Do you have a spouse or partner who lives in this household?”* | *“Yes” or “No”.* | - | “Yes” or “No”. |
| Geographic region | Federation unity | States of Brazil and the Federal District. | Region made up of different territories with certain common characteristics (29). | “North”; “Northeast”; “Southeast”; “South”; or “Midwest”. |
| Area of residence | Type of census situation | *“Urban” or “Rural’.* | - | “Urban” or “Rural” (29). |
| Tobacco use | *“Do you currently smoke any tobacco products?”* | *“Yes; daily”; “Yes; less than daily”;* or *“I don't currently smoke”.* | - | “Non-smoker”; “Former smoker”; or “Current smoker” (30). |
|  | *“And in the past, did you smoke any tobacco products?”* (for those who answered “no” to the previous question) | *“Yes” or “No”.* |  |  |
| Alcohol abuse | *“In the past 30 days, have you consumed five or more alcoholic drinks on a single occasion (a standard drink was defined as the equivalent of a can of beer, a glass of wine, a shot of cachaça, whiskey or any other distilled alcoholic beverage)?”* | *“Yes” or “No”.* | The minimum consumption of five alcoholic drinks on a single occasion in the past 30 days (31)*.* | “Yes” or “No”. |
| Leisure-time physical activity | *“In the last twelve months, did you practice any type of physical activity or sport? (do not consider physiotherapy)”* | *“Yes” or “No”.* | Physically active individuals who reported practicing at least 150 minutes per week of light or moderate intensity, or 75 minutes per week of vigorous-intensity physical activity (32).  Classified as light or moderate intensity practices:  Walking, treadmill walking, hydrogymnastics, localized gymnastics/pilates/stretching or yoga, swimming, martial arts and wrestling, bicycle or stationary bicycle, volleyball, and dance class, among others (32).  Classified as vigorous physical activities:  Running, treadmill running, weight training, aerobics/spinning/step/jump, soccer, basketball, and tennis (32). | “Active” or “Inactive”. |
|  | *“How many days a week do you (did you) practice physical activities or sports?”* | *“Number of days”* or *“Never or less than once a week”.* |  |  |
|  | *“In general, on the days you do (you did) physical activities or sports, how long does (did) this activity last?”* | In hours and minutes. |  |  |
|  | *“What physical exercise or sport do you (did you) practice most often?”* | *“Walking”; “Treadmill walking”; “Running”; “Treadmill running”; “Weight training”; “Aerobics/spinning/step/jump”; “Hydrogymnastics”; “Localized gymnastics/Pilates/stretching or yoga”; “Swimming”; “Martial arts and wrestling”; “Bicycle or stationary bicycle”; “Soccer”; “Basketball”; “Volleyball”; “Tennis” “Dance class”; or “Others”.* |  |  |
| Recommended consumption of fruits and vegetables | *“How many days a week do you usually eat fruit?”,* | *“Number of days”* or *“Never or less than once a week”.* | Consumption of at least 25 servings of fruits (including juice) and vegetables per week. These servings are equivalent to the daily consumption of approximately five servings of fruits and vegetables (33). | “Yes” or “No”. |
|  | *“In general, how many times a day do you eat fruit?”* | *“Once a day”; “Twice a day”; or “Three times or more a day”.* |  |  |
|  | *“How many days a week do you usually drink natural fruit juice (including frozen fruit pulp)?”,* | *“Number of days”* or *“Never or less than once a week”.* |  |  |
|  | *“How many days a week do you usually eat at least one type of vegetable (excluding potatoes, cassava, or yams) such as lettuce, tomato, cabbage, carrots, chayote, eggplant, zucchini?”;* | *“Number of days”* or *“Never or less than once a week”.* |  |  |
|  | *“In general, do you usually eat this type of vegetable?”* | *“Once a day (at lunch or dinner)”; “twice a day (at lunch or dinner);” or “three times or more per day*”. |  |  |
| Ultra-processed food consumption | *“Yesterday, did you drink or eat: Soda; Fruit juice in a box or can or powdered soft drink; Chocolate milk beverages or flavored yogurt; Packaged snacks or salted crackers; Sweet or stuffed biscuit/cookie or packaged cake; Ice cream, chocolate, jelly, flan or other industrialized desserts; wiener, sausage, mortadella or ham; Loaf bread, hot dog or hamburger bread; Margarine, mayonnaise, ketchup or other industrialized sauces; Instant noodles, packaged soup, frozen lasagna, or other ready-to-eat frozen food”* | *“Yes” or “No”* for each food group. | Consumption of five or more ultra-processed food groups on the day before the survey which was calculated by summing the positive responses to consumption among the ultra-processed food subgroups (33). | “Yes” or “No”. |
| Regular consumption of soft drinks or artificial juices | *“How many days a week do you usually drink juice in a box or can, or powdered soft drink?”* | *“Number of days”* or *“Never or less than once a week”.* | Consumption of soft drinks or artificial juices at least five days a week (33). | “Yes” or “No”. |
|  | *“How many days a week do you usually drink soda?”* | *“Number of days”* or *“Never or less than once a week”.* |  |  |
| Nutritional status | *“Do you know your weight?”* | In kilograms. | Nutritional status was classified using Body Mass Index (BMI) as recommended by the World Health Organization (WHO) (34). The BMI was calculated by the ratio between weight (kg) and height squared (m²). | “Underweight (<18.5 kg/m²)”; “Normal weight (18.6 to 24.9 kg/m²)”; “Overweight (25.0 to 29.9 kg/m²)”; and “Obese (≥30 kg/m²)”. |
|  | *“Do you know your height?”* | In centimeters. |  |  |

**Supplementary Table 3.** Bivariate regression analysis of factors associated with multimorbidity in the total sample. National Health Survey, Brazil, 2019

| **Variables** | **Multimorbidity**  **(n=88,531)** | | | | |
| --- | --- | --- | --- | --- | --- |
|  | **%†** | **95% CI** | **PR** | **95% CI** | **p-Value\|\|** |
| **Age group (years)** |  |  |  |  |  |
| 18-24 | 8.3 | 7.3-9.4 | 1.00 |  |  |
| 25-39 | 13.8 | 13.1-14.6 | 1.66 | 1.45-1.91 | <0.001 |
| 40-59 | 34.1 | 33.2-35.1 | 4.11 | 3.61-4.68 | <0.001 |
| ≥60 | 56.5 | 55.4-57.6 | 6.80 | 5.99-7.71 | <0.001 |
| **Self-declared race/skin color*** |  |  |  |  |  |
| White | 32.2 | 31.4-33.1 | 1.20 | 1.15-1.24 | <0.001 |
| Brown | 27.0 | 26.2-27.7 | 1.00 |  |  |
| Black | 28.1 | 26.6-29.6 | 1.04 | 0.98-1.10 | <0.001 |
| Others (yellow or indigenous) | 32.0 | 27.1-37.4 | 1.19 | 1.01-1.40 | <0.001 |
| **Education** |  |  |  |  |  |
| No education or incomplete middle school | 40.3 | 39.3-41.2 | 1.46 | 1.39-1.54 | <0.001 |
| Complete middle school or incomplete high school | 24.1 | 22.9-25.5 | 0.88 | 0.82-0.94 | <0.001 |
| Complete high school or incomplete higher education | 21.7 | 20.9-22.6 | 0.79 | 0.74-0.84 | <0.001 |
| Complete higher education or more | 27.6 | 26.3-28.9 | 1.00 |  |  |
| **Living with a spouse/partner** |  |  |  |  |  |
| No | 29.2 | 28.4-30.1 | 1.00 |  |  |
| Yes | 29.6 | 28.9-30.3 | 1.0.1 | 0.98-1.05 | 0.479 |
| **Geographic region** |  |  |  |  |  |
| North | 21.1 | 20.1-22.2 | 1.00 |  |  |
| Northeast | 26.3 | 25.4-27.1 | 1.24 | 1.17-1.32 | <0.001 |
| Southeast | 32.1 | 31.1-33.2 | 1.52 | 1.43-1.62 | <0.001 |
| South | 33.0 | 31.7-34.3 | 1.56 | 1.47-1.66 | <0.001 |
| Midwest | 26.9 | 25.6-28.2 | 1.27 | 1.19-1.37 | <0.001 |
| **Area of residence** |  |  |  |  |  |
| Urban | 30.0 | 29.4-30.6 | 1.16 | 1.10-1.21 | <0.001 |
| Rural | 26.0 | 24.9-27.1 | 1.00 |  |  |
| **Tobacco use** |  |  |  |  |  |
| Non-smoker | 25.1 | 24.4-25.8 | 1.00 |  |  |
| Former smoker | 39.9 | 38.8-41.1 | 1.59 | 1.53-1.66 | <0.001 |
| Smoker | 28.3 | 26.8-29.8 | 1.13 | 1.06-1.20 | <0.001 |
| **Alcohol abuse** |  |  |  |  |  |
| No | 31.4 | 30.8-32.1 | 1.00 |  |  |
| Yes | 19.7 | 18.7-20.8 | 0.63 | 0.59-0.66 | <0.001 |
| **Leisure-time physical activity** |  |  |  |  |  |
| Active | 23.7 | 22.8-24.6 | 1.00 |  |  |
| Inactive | 31.9 | 31.2-32.6 | 1.35 | 1.29-1.41 | <0.001 |
| **Recommended fruit and vegetable consumption** |  |  |  |  |  |
| No | 28.3 | 27.7-28.9 | 0.76 | 0.73-0.80 | <0.001 |
| Yes | 37.0 | 35.5-38.6 | 1.00 |  |  |
| **Ultra-processed food consumption** |  |  |  |  |  |
| No | 30.9 | 30.3-31.5 | 1.00 |  |  |
| Yes | 20.9 | 19.6-22.2 | 0.68 | 0.63-0.72 | <0.001 |
| **Regular consumption of soft drinks and/or artificial juices** | | | | | |
| No | 31.9 | 31.2-32.5 | 1.00 |  |  |
| Yes | 24.5 | 23.5-25.5 | 0.77 | 0.73-0.81 | <0.001 |
| **Nutritional status** |  |  |  |  |  |
| Low weight | 24.1 | 20.6-28.1 | 1.05 | 0.90-1.23 | 0.536 |
| Normal weight | 23.0 | 22.2-23.8 | 1.00 |  |  |
| Overweight | 31.2 | 30.3-32.1 | 1.36 | 1.30-1.42 | <0.001 |
| Obese | 40.0 | 38.7-41.3 | 1.74 | 1.66-1.83 | <0.001 |

**Notes:** 95% CI: 95% Confidence Interval; PR: Prevalence Ratio.

**Missing data*: 9 (6 men and 3 women).

†The prevalence values are weighted by the complex sample.

|| Wald chi-square test.

**Supplementary Table 4.** Bivariate regression analysis of factors associated with multimorbidity in men. National Health Survey, Brazil, 2019

| **Variables** | **Multimorbidity**  **(n=41,662)** | | | | |
| --- | --- | --- | --- | --- | --- |
|  | **%†** | **95% CI** | **PR** | **95% CI** | **p-Value\|\|** |
| **Age group (years)** |  |  |  |  |  |
| 18-24 | 6.4 | 5.1-8.0 | 1.00 |  |  |
| 25-39 | 10.0 | 9.1-11.0 | 1.56 | 1.21-2.01 | <0.001 |
| 40-59 | 26.3 | 25.1-27.6 | 4.12 | 3.26-5.21 | <0.001 |
| ≥60 | 47.5 | 45.9-49.1 | 7.43 | 5.91-9.34 | <0.001 |
| **Self-reported race/skin color*** |  |  |  |  |  |
| White | 25.4 | 24.2-26.5 | 1.23 | 1.15-1.32 | <0.001 |
| Brown | 20.6 | 19.5-21.6 | 1.00 |  |  |
| Black | 20.2 | 18.4-22.2 | 0.99 | 0.89-1.09 | 0.778 |
| Others (yellow or indigenous) | 26.9 | 19.8-35.4 | 1.31 | 0.97-1.77 | 0.077 |
| **Education** |  |  |  |  |  |
| No education or incomplete middle school | 29.7 | 28.5-31.0 | 1.18 | 1.08-1.29 | <0.001 |
| Complete middle school or incomplete high school | 17.4 | 15.7-19.1 | 0.69 | 0.61-0.78 | <0.001 |
| Complete high school or incomplete higher education | 16.8 | 15.7-17.9 | 0.67 | 0.60-0.74 | <0.001 |
| Complete higher education or more | 25.1 | 23.2-27.2 | 1.00 |  |  |
| **Living with a spouse/partner** |  |  |  |  |  |
| No | 16.5 | 15.4-17.6 | 1.00 |  |  |
| Yes | 25.5 | 24.6-26.5 | 1.55 | 1.44-1.67 | <0.001 |
| **Geographic region** |  |  |  |  |  |
| North | 16.0 | 14.8-17.3 | 1.00 |  |  |
| Northeast | 19.2 | 18.1-20.3 | 1.20 | 1.09-1.32 | <0.001 |
| Southeast | 25.2 | 23.8-26.6 | 1.57 | 1.43-1.73 | <0.001 |
| South | 26.4 | 24.8-28.0 | 1.65 | 1.49-1.82 | <0.001 |
| Midwest | 20.3 | 18.5-22.3 | 1.27 | 1.13-1.44 | <0.001 |
| **Area of residence** |  |  |  |  |  |
| Urban | 23.2 | 22.4-24.1 | 1.19 | 1.10-1.27 | <0.001 |
| Rural | 19.6 | 18.4-20.8 | 1.00 |  |  |
| **Tobacco use** |  |  |  |  |  |
| Non-smoker | 17.6 | 16.8-18.5 | 1.00 |  |  |
| Former smoker | 34.5 | 32.9-36.1 | 1.96 | 1.83-2.09 | <0.001 |
| Smoker | 21.0 | 19.3-22.9 | 1.20 | 1.09-1.32 | <0.001 |
| **Alcohol abuse** |  |  |  |  |  |
| No | 24.5 | 23.6-25.3 | 1.00 |  |  |
| Yes | 17.6 | 16.4-18.8 | 0.72 | 0.66-0.78 | <0.001 |
| **Leisure-time physical activity** |  |  |  |  |  |
| Active | 25.4 | 24.5-26.3 | 1.00 |  |  |
| Inactive | 17.4 | 16.3-18.6 | 1.45 | 1.35-1.56 | <0.001 |
| **Recommended fruit and vegetable consumption** |  |  |  |  |  |
| No | 22.0 | 21.2-22.7 | 0.76 | 0.69-0.84 | <0.001 |
| Yes | 28.9 | 26.3-31.6 | 1.00 |  |  |
| **Ultra-processed food consumption** |  |  |  |  |  |
| No | 24.0 | 23.2-24.8 | 1.00 |  |  |
| Yes | 15.7 | 14.1-17.3 | 0.65 | 0.59-0.73 | <0.001 |
| **Regular consumption of soft drinks and/or artificial juices** |  |  |  |  |  |
| No | 24.7 | 23.9-25.6 | 1.00 |  |  |
| Yes | 19.2 | 17.9-20.5 | 0.77 | 0.72-0.83 | <0.001 |
| **Nutritional status** |  |  |  |  |  |
| Low weight | 15.7 | 11.6-20.8 | 0.92 | 0.68-1.23 | 0.562 |
| Normal weight | 17.1 | 16.1-18.2 | 1.00 |  |  |
| Overweight | 24.5 | 23.3-25.6 | 1.43 | 1.32-1.55 | <0.001 |
| Obese | 30.8 | 28.9-32.7 | 1.80 | 1.65-1.96 | <0.001 |

**Notes:** 95% CI: 95% Confidence Interval; PR: Prevalence Ratio.

**Missing data*: 6.

†The prevalence values are weighted by the complex sample.

|| Wald chi-square test.

**Supplementary Table 5.** Bivariate regression analysis of factors associated with multimorbidity in women. National Health Survey, Brazil, 2019

| **Variables** | **Multimorbidity**  **(n=46,869)** | | | | |
| --- | --- | --- | --- | --- | --- |
|  | **%†** | **95% CI** | **PR** | **95% CI** | **p-Value\|\|** |
| **Age group (years)** |  |  |  |  |  |
| 18-24 | 10.2 | 8.8-11.9 | 1.00 |  |  |
| 25-39 | 17.4 | 16.3-18.6 | 1.70 | 1.44-2.00 | <0.001 |
| 40-59 | 40.9 | 39.6-42.2 | 3.99 | 3.42-4.67 | <0.001 |
| ≥60 | 63.4 | 61.9-64.8 | 6.18 | 5.30-7.22 | <0.001 |
| **Self-reported race/skin color*** |  |  |  |  |  |
| White | 38.1 | 36.9-39.4 | 1.16 | 1.11-1.22 | <0.001 |
| Brown | 32.7 | 31.6-33.8 | 1.00 |  |  |
| Black | 35.1 | 33.0-37.2 | 1.07 | 1.00-1.14 | 0.043 |
| Others (yellow or indigenous) | 37.3 | 30.9-44.1 | 1.14 | 0.95-1.36 | 0.158 |
| **Education** |  |  |  |  |  |
| No education or incomplete middle school | 49.9 | 48.6-51.3 | 1.70 | 1.60-1.82 | <0.001 |
| Complete middle school or incomplete high school | 31.1 | 29.3-33.0 | 1.06 | 0.98-1.16 | 0.158 |
| Complete high school or incomplete higher education | 26.0 | 24.8-27.3 | 0.89 | 0.83-0.96 | 0.002 |
| Complete higher education or more | 29.3 | 27.6-31.1 | 1.00 |  |  |
| **Living with a spouse/partner** |  |  |  |  |  |
| No | 37.2 | 36.1-38.4 | 1.00 |  |  |
| Yes | 34.0 | 32.9-35.0 | 0.91 | 0.88-0.95 | <0.001 |
| **Geographic region** |  |  |  |  |  |
| North | 25.9 | 24.4-27.5 | 1.00 |  |  |
| Northeast | 32.4 | 31.2-33.6 | 1.25 | 1.17-1.34 | <0.001 |
| Southeast | 38.2 | 36.7-39.8 | 1.48 | 1.37-1.59 | <0.001 |
| South | 38.9 | 37.0-40.8 | 1.50 | 1.39-1.62 | <0.001 |
| Midwest | 32.8 | 31.1-34.6 | 1.27 | 1.17-1.37 | <0.001 |
| **Area of residence** |  |  |  |  |  |
| Urban | 35.7 | 34.8-36.6 | 1.08 | 1.02-1.13 | 0.007 |
| Rural | 33.2 | 31.7-34.8 | 1.00 |  |  |
| **Tobacco use** |  |  |  |  |  |
| Non-smoker | 31.0 | 30.0-32.0 | 1.00 |  |  |
| Former smoker | 44.8 | 43.3-46.4 | 1.45 | 1.38-1.51 | <0.001 |
| Smoker | 38.8 | 36.5-41.3 | 1.25 | 1.117-1.34 | <0.001 |
| **Alcohol abuse** |  |  |  |  |  |
| No | 36.5 | 35.6-37.3 | 1.00 |  |  |
| Yes | 25.1 | 23.0-27.3 | 0.69 | 0.63-0.75 | <0.001 |
| **Leisure-time physical activity** |  |  |  |  |  |
| Active | 37.1 | 36.2-38.0 | 1.00 |  |  |
| Inactive | 30.8 | 29.3-32.3 | 1.21 | 1.14-1.27 | <0.001 |
| **Recommended fruit and vegetable consumption** |  |  |  |  |  |
| No | 34.3 | 33.4-35.1 | 0.82 | 0.78-0.86 | <0.001 |
| Yes | 41.8 | 39.9-43.7 | 1.00 |  |  |
| **Ultra-processed food consumption** |  |  |  |  |  |
| No | 36.8 | 35.9-37.6 | 1.00 |  |  |
| Yes | 26.4 | 24.4-28.5 | 0.72 | 0.66-0.78 | <0.001 |
| **Regular consumption of soft drinks and/or artificial juices** | | | | | |
| No | 37.4 | 36.5-38.3 | 1.00 |  |  |
| Yes | 30.5 | 29.0-32.1 | 0.82 | 0.77-0.86 | <0.001 |
| **Nutritional status** |  |  |  |  |  |
| Low weight | 28.7 | 24.0-34.0 | 1.02 | 0.86-1.23 | 0.792 |
| Normal weight | 28.1 | 26.9-29.3 | 1.00 |  |  |
| Overweight | 38.3 | 37.0-39.7 | 1.37 | 1.29-1.44 | <0.001 |
| Obese | 46.9 | 45.2-48.6 | 1.67 | 1.58-1.77 | <0.001 |

**Notes:** 95% CI: 95% Confidence Interval; PR: Prevalence Ratio.

**Missing data*: 3.

†The prevalence values are weighted by the complex sample.

|| Wald chi-square test.

**Supplementary Table 6.** Interaction terms of multiple regression analysis of factors associated with multimorbidity in the total sample. National Health Survey, Brazil, 2019

| **Terms of interaction** | **APR** | **95% CI** | **p-Value\|\|** | **RD** |
| --- | --- | --- | --- | --- |
| **Age group (years) and Geographic region** |  |  |  |  |
| 25-39 x Northeast | 0.98 | 0.67 - 1.44 | 0.928 | 1.91 |
| 25-39 x Southeast | 0.88 | 0.60 - 1.29 | 0.510 | 2.38 |
| 25-39 x South | 0.67 | 0.45 - 0.99 | **0.047** | **2.28** |
| 25-39 x Midwest | 0.78 | 0.50 - 1.23 | 0.286 | 1.99 |
| 40-59 x Northeast | 1.16 | 0.81 - 1.66 | 0.424 | 5.08 |
| 40-59 x Southeast | 0.80 | 0.56 - 1.15 | 0.225 | 4.85 |
| 40-59 x South | 0.65 | 0.45 - 0.93 | **0.020** | **4.97** |
| 40-59 x Midwest | 0.70 | 0.45 - 1.10 | 0.125 | 4.01 |
| ≥60 x Northeast | 1.13 | 0.79 - 1.61 | 0.515 | 8.05 |
| ≥60 x Southeast | 0.73 | 0.51 - 1.05 | 0.088 | 7.21 |
| ≥60 x South | 0.58 | 0.41 - 0.84 | **0.003** | **7.21** |
| ≥60 x Midwest | 0.75 | 0.48 - 1.17 | 0.207 | 6.98 |
| **Education and e Self-reported race/skin color** |  |  |  |  |
| No education or incomplete middle school x White | 1.04 | 0.93 - 1.16 | 0.525 | 1.08 |
| No education or incomplete middle school x Black | 1.31 | 1.05 - 1.62 | **0.014** | **1.16** |
| No education or incomplete middle school x Others (yellow or indigenous) | 1.14 | 0.83 - 1.58 | 0.415 | 0.92 |
| Complete middle school or incomplete high school x White | 1.14 | 0.97 - 1.34 | 0.101 | 1.00 |
| Complete middle school or incomplete high school x Black | 1.54 | 1.19 - 1.99 | **0.001** | **1.15** |
| Complete middle school or incomplete high school x Others (yellow or indigenous) | 1.37 | 0.95 - 1.97 | 0.090 | 0.93 |
| Complete high school or incomplete higher education x White | 1.18 | 1.03 - 1.35 | **0.014** | **1.09** |
| Complete high school or incomplete higher education x Black | 1.40 | 1.11 - 1.77 | **0.005** | **1.10** |
| Complete high school or incomplete higher education x Others (yellow or indigenous) | 1.60 | 1.05 - 2.44 | **0.027** | **1.14** |
| **Geographic region and e Self-reported race/skin color** |  |  |  |  |
| Northeast x White | 0.88 | 0.79 - 0.99 | **0.040** | **1.06** |
| Northeast x Black | 0.83 | 0.70 - 0.97 | **0.018** | **0.85** |
| Northeast x Others (yellow or indigenous) | 1.25 | 0.84 - 1.86 | 0.267 | 1.17 |
| Southeast x White | 0.90 | 0.8 - 1.02 | 0.098 | 1.51 |
| Southeast x Black | 0.79 | 0.67 - 0.93 | **0.004** | **1.12** |
| Southeast x Others (yellow or indigenous) | 1.08 | 0.73 - 1.60 | 0.696 | 1.40 |
| South x White | 0.85 | 0.74 - 0.98 | **0.026** | **1.79** |
| South x Black | 0.95 | 0.77 - 1.17 | 0.606 | 1.70 |
| South x Others (yellow or indigenous) | 0.64 | 0.38 - 1.09 | 0.100 | 1.04 |
| Midwest x White | 0.87 | 0.76 – 1.00 | 0.053 | 1.37 |
| Midwest x Black | 0.72 | 0.58 - 0.89 | **0.002** | **0.97** |
| Midwest x Others (yellow or indigenous) | 1.12 | 0.74 - 1.70 | 0.599 | 1.37 |
| **Education and Geographic region** |  |  |  |  |
| No education or incomplete middle school x Northeast | 0.89 | 0.76 - 1.03 | 0.125 | 0.99 |
| No education or incomplete middle school x Southeast | 1.11 | 0.95 - 1.30 | 0.200 | 1.72 |
| No education or incomplete middle school x South | 1.18 | 0.99 - 1.42 | 0.064 | 2.30 |
| No education or incomplete middle school x Midwest | 1.15 | 0.96 - 1.38 | 0.135 | 1.68 |
| Complete middle school or incomplete high school x Northeast | 0.88 | 0.72 - 1.09 | 0.236 | 0.83 |
| Complete middle school or incomplete high school x Southeast | 1.09 | 0.88 - 1.34 | 0.450 | 1.43 |
| Complete middle school or incomplete high school x South | 1.02 | 0.81 - 1.29 | 0.844 | 1.68 |
| Complete middle school or incomplete high school x Midwest | 1.10 | 0.87 - 1.40 | 0.425 | 1.36 |
| Complete high school or incomplete higher education x Northeast | 1.00 | 0.84 - 1.19 | 0.964 | 0.99 |
| Complete high school or incomplete higher education x Southeast | 1.11 | 0.93 - 1.32 | 0.241 | 1.53 |
| Complete high school or incomplete higher education x South | 1.10 | 0.90 - 1.35 | 0.348 | 1.90 |
| Complete high school or incomplete higher education x Midwest | 1.20 | 0.99 - 1.46 | 0.060 | 1.56 |
| **Ultra-processed food consumption and Recommended fruit and vegetable consumption** |  |  |  |  |
| Yes x No | 0.87 | 0.75 - 1.01 | 0.060 | 0.83 |

**Notes:** 95% CI: 95% Confidence Interval; APR: Adjusted Prevalence Ratio; RD: Risk difference.

*Wald chi-square test.

**Supplementary Table 7.** Interaction terms of multiple regression analysis of factors associated with multimorbidity in men. National Health Survey, Brazil, 2019

| **Terms of interaction** | **APR** | **95% CI** | **p-Value\|\|** | **RD** |
| --- | --- | --- | --- | --- |
| **Age group (years) and Geographic region** |  |  |  |  |
| 25-39 x Northeast | 0.91 | 0.47 - 1.74 | 0.772 | 1.72 |
| 25-39 x Southeast | 0.83 | 0.44 - 1.58 | 0.570 | 1.90 |
| 25-39 x South | 0.95 | 0.50 - 1.82 | 0.886 | 2.39 |
| 25-39 x Midwest | 1.21 | 0.49 - 2.98 | 0.673 | 1.88 |
| 40-59 x Northeast | 1.14 | 0.62 - 2.08 | 0.678 | 4.91 |
| 40-59 x Southeast | 0.84 | 0.46 - 1.52 | 0.561 | 4.39 |
| 40-59 x South | 0.95 | 0.52 - 1.74 | 0.876 | 5.47 |
| 40-59 x Midwest | 1.02 | 0.43 - 2.42 | 0.972 | 3.62 |
| ≥60 x Northeast | 1.02 | 0.57 - 1.84 | 0.938 | 8.08 |
| ≥60 x Southeast | 0.76 | 0.43 - 1.36 | 0.361 | 7.31 |
| ≥60 x South | 0.82 | 0.46 - 1.46 | 0.496 | 8.68 |
| ≥60 x Midwest | 1.01 | 0.43 - 2.38 | 0.978 | 6.60 |
| **Education and e Self-reported race/skin color** |  |  |  |  |
| No education or incomplete middle school x White | 1.01 | 0.83 - 1.23 | 0.948 | 1.07 |
| No education or incomplete middle school x Black | 1.13 | 0.79 - 1.60 | 0.504 | 0.92 |
| No education or incomplete middle school x Others (yellow or indigenous) | 1.41 | 0.79 - 2.52 | 0.249 | 0.95 |
| Complete middle school or incomplete high school x White | 1.22 | 0.92 - 1.61 | 0.166 | 1.05 |
| Complete middle school or incomplete high school x Black | 1.36 | 0.87 - 2.10 | 0.175 | 0.90 |
| Complete middle school or incomplete high school x Others (yellow or indigenous) | 2.21 | 1.18 - 4.15 | **0.013** | **1.20** |
| Complete high school or incomplete higher education x White | 1.12 | 0.89 - 1.40 | 0.324 | 0.88 |
| Complete high school or incomplete higher education x Black | 1.32 | 0.88 - 1.97 | 0.174 | 0.80 |
| Complete high school or incomplete higher education x Others (yellow or indigenous) | 2.65 | 1.35 - 5.17 | **0.004** | **1.32** |
| **Geographic region and e Self-reported race/skin color** |  |  |  |  |
| Northeast x White | 0.77 | 0.63 - 0.94 | **0.011** | **1.26** |
| Northeast x Black | 0.96 | 0.73 - 1.25 | 0.746 | 1.21 |
| Northeast x Others (yellow or indigenous) | 1.01 | 0.49 - 2.11 | 0.971 | 1.05 |
| Southeast x White | 0.74 | 0.6 - 0.91 | **0.005** | **1.47** |
| Southeast x Black | 0.78 | 0.60 - 1.02 | 0.068 | 1.19 |
| Southeast x Others (yellow or indigenous) | 0.82 | 0.40 - 1.69 | 0.592 | 1.03 |
| South x White | 0.69 | 0.55 - 0.87 | **0.001** | **1.51** |
| South x Black | 0.84 | 0.59 - 1.21 | 0.353 | 1.41 |
| South x Others (yellow or indigenous) | 0.43 | 0.19 - 0.98 | **0.044** | **0.59** |
| Midwest x White | 0.72 | 0.57 - 0.90 | **0.005** | **0.97** |
| Midwest x Black | 0.65 | 0.46 - 0.91 | **0.012** | **0.67** |
| Midwest x Others (yellow or indigenous) | 0.85 | 0.40 - 1.81 | 0.676 | 0.73 |
| **Education and Geographic region** |  |  |  |  |
| No education or incomplete middle school x Northeast | 0.8 | 0.61 - 1.07 | 0.128 | 0.89 |
| No education or incomplete middle school x Southeast | 1.15 | 0.87 - 1.52 | 0.322 | 1.55 |
| No education or incomplete middle school x South | 1.09 | 0.81 - 1.48 | 0.568 | 1.62 |
| No education or incomplete middle school x Midwest | 1.21 | 0.86 - 1.70 | 0.273 | 1.11 |
| Complete middle school or incomplete high school x Northeast | 0.82 | 0.56 - 1.19 | 0.289 | 0.74 |
| Complete middle school or incomplete high school x Southeast | 1.19 | 0.82 - 1.72 | 0.350 | 1.31 |
| Complete middle school or incomplete high school x South | 0.8 | 0.53 - 1.19 | 0.272 | 0.97 |
| Complete middle school or incomplete high school x Midwest | 1.16 | 0.76 - 1.75 | 0.493 | 0.86 |
| Complete high school or incomplete higher education x Northeast | 0.99 | 0.72 - 1.37 | 0.958 | 0.82 |
| Complete high school or incomplete higher education x Southeast | 1.28 | 0.93 - 1.77 | 0.130 | 1.28 |
| Complete high school or incomplete higher education x South | 1.13 | 0.79 - 1.62 | 0.512 | 1.25 |
| Complete high school or incomplete higher education x Midwest | 1.26 | 0.87 - 1.82 | 0.221 | 0.86 |
| **Ultra-processed food consumption and Recommended fruit and vegetable consumption** |  |  |  |  |
| Yes x No | 1.07 | 0.82 - 1.39 | 0.636 | 0.80 |

**Notes:** 95% CI: 95% Confidence Interval; APR: Adjusted Prevalence Ratio; RD: Risk difference.

*Wald chi-square test.

**Supplementary Table 8.** Interaction terms of multiple regression analysis of factors associated with multimorbidity in women. National Health Survey, Brazil, 2019

| **Terms of interaction** | **APR** | **95% CI** | **p-Value\|\|** | **RD** |
| --- | --- | --- | --- | --- |
| **Age group (years) and Geographic region** |  |  |  |  |
| 25-39 x Northeast | 1.04 | 0.66 - 1.64 | 0.864 | 2.08 |
| 25-39 x Southeast | 0.92 | 0.57 - 1.47 | 0.723 | 2.70 |
| 25-39 x South | 0.55 | 0.34 - 0.89 | **0.014** | **2.22** |
| 25-39 x Midwest | 0.62 | 0.35 - 1.09 | 0.094 | 2.06 |
| 40-59 x Northeast | 1.18 | 0.77 - 1.81 | 0.453 | 5.25 |
| 40-59 x Southeast | 0.78 | 0.50 - 1.22 | 0.280 | 5.09 |
| 40-59 x South | 0.52 | 0.33 - 0.81 | **0.004** | **4.67** |
| 40-59 x Midwest | 0.57 | 0.34 - 0.98 | **0.041** | **4.22** |
| ≥60 x Northeast | 1.21 | 0.78 - 1.86 | 0.395 | 8.08 |
| ≥60 x Southeast | 0.72 | 0.46 - 1.13 | 0.154 | 7.05 |
| ≥60 x South | 0.48 | 0.31 - 0.76 | **0.001** | **6.47** |
| ≥60 x Midwest | 0.64 | 0.37 - 1.10 | 0.106 | 7.11 |
| **Education and e Self-reported race/skin color** |  |  |  |  |
| No education or incomplete middle school x White | 1.06 | 0.93 - 1.22 | 0.394 | 1.10 |
| No education or incomplete middle school x Black | 1.39 | 1.08 - 1.80 | **0.012** | **1.35** |
| No education or incomplete middle school x Others (yellow or indigenous) | 1.01 | 0.70 - 1.46 | 0.972 | 0.93 |
| Complete middle school or incomplete high school x White | 1.12 | 0.92 - 1.35 | 0.257 | 1.00 |
| Complete middle school or incomplete high school x Black | 1.64 | 1.20 - 2.24 | **0.002** | **1.37** |
| Complete middle school or incomplete high school x Others (yellow or indigenous) | 1.06 | 0.68 - 1.65 | 0.786 | 0.84 |
| Complete high school or incomplete higher education x White | 1.23 | 1.05 - 1.45 | **0.012** | **1.04** |
| Complete high school or incomplete higher education x Black | 1.43 | 1.08 - 1.90 | **0.013** | **1.13** |
| Complete high school or incomplete higher education x Others (yellow or indigenous) | 1.07 | 0.66 - 1.75 | 0.776 | 0.80 |
| **Geographic region and e Self-reported race/skin color** |  |  |  |  |
| Northeast x White | 0.95 | 0.83 - 1.10 | 0.496 | 0.98 |
| Northeast x Black | 0.74 | 0.61 - 0.90 | **0.002** | **0.71** |
| Northeast x Others (yellow or indigenous) | 1.40 | 0.91 - 2.15 | 0.121 | 1.27 |
| Southeast x White | 1.00 | 0.87 - 1.16 | 0.964 | 1.51 |
| Southeast x Black | 0.77 | 0.64 - 0.94 | **0.008** | **1.09** |
| Southeast x Others (yellow or indigenous) | 1.22 | 0.83 - 1.79 | 0.309 | 1.63 |
| South x White | 0.96 | 0.81 - 1.14 | 0.647 | 1.99 |
| South x Black | 1.00 | 0.79 - 1.26 | 0.975 | 1.94 |
| South x Others (yellow or indigenous) | 0.82 | 0.45 - 1.47 | 0.505 | 1.51 |
| Midwest x White | 0.99 | 0.84 - 1.16 | 0.861 | 1.69 |
| Midwest x Black | 0.75 | 0.58 - 0.96 | **0.022** | **1.20** |
| Midwest x Others (yellow or indigenous) | 1.31 | 0.82 - 2.07 | 0.257 | 1.98 |
| **Education and Geographic region** |  |  |  |  |
| No education or incomplete middle school x Northeast | 0.91 | 0.76 - 1.10 | 0.337 | 1.05 |
| No education or incomplete middle school x Southeast | 1.09 | 0.90 - 1.32 | 0.396 | 1.85 |
| No education or incomplete middle school x South | 1.23 | 0.99 - 1.52 | 0.067 | 2.87 |
| No education or incomplete middle school x Midwest | 1.11 | 0.89 - 1.39 | 0.358 | 2.13 |
| Complete middle school or incomplete high school x Northeast | 0.90 | 0.71 - 1.15 | 0.404 | 0.90 |
| Complete middle school or incomplete high school x Southeast | 0.10 | 0.81 - 1.33 | 0.781 | 0.15 |
| Complete middle school or incomplete high school x South | 1.20 | 0.91 - 1.59 | 0.203 | 2.41 |
| Complete middle school or incomplete high school x Midwest | 1.07 | 0.79 - 1.43 | 0.670 | 1.77 |
| Complete high school or incomplete higher education x Northeast | 0.98 | 0.79 - 1.20 | 0.822 | 0.92 |
| Complete high school or incomplete higher education x Southeast | 1.03 | 0.83 - 1.27 | 0.792 | 1.42 |
| Complete high school or incomplete higher education x South | 1.10 | 0.86 - 1.40 | 0.468 | 2.09 |
| Complete high school or incomplete higher education x Midwest | 1.16 | 0.92 - 1.47 | 0.218 | 1.82 |
| **Ultra-processed food consumption and Recommended fruit and vegetable consumption** |  |  |  |  |
| Yes x No | 0.80 | 0.67 - 0.94 | **0.009** | **0.83** |

**Notes:** 95% CI: 95% Confidence Interval; APR: Adjusted Prevalence Ratio; RD: Risk difference.

*Wald chi-square test.
